# Accelerating Research and Policy on PFAS in India

**DOI:** 10.1097/EE9.0000000000000199

**Published:** 2022-02-22

**Authors:** Ankan Mukherjee Das, Rajiv Janardhanan

**Affiliations:** Laboratory of Disease Dynamics and Molecular Epidemiology, Amity Institute of Public Health, Amity University, Noida, India

The roadmap introduced by the US Environmental Protection Agency^1^ directed toward accelerated research, restriction, and cleanup of per- and polyfluoroalkyl substances (PFAS) is a strong and well-timed initiative that encompasses to evaluate and contain their toxic effects on human health and ecology through effective interventions. Recent regulations in Denmark^2^ prohibiting use of paper and cardboard food contact materials, and articles containing PFAS is also an indication of increasing public awareness and concerns regarding their hazardous impact on public health. Learning from this, although in 2020, the Bureau of Indian Standards adopted the International Standards Organizations criterion for sampling and testing of perfluorooctanoic acid and perfluorooctane sulfonate, further initiatives are expeditiously needed to tackle the unregulated and indiscriminate use of ubiquitous everyday toxic chemicals such as phthalates and PFAS in consumer products used by children and adults, including single-use plastics, personal-care and cosmetics, processed food, and packaging, which are the major source of contact with significant adverse effects on human health and environment.

Despite the Stockholm Convention on Persistent Organic Pollutants included PFAS in the restricted list of chemicals for effective monitoring and control, these are indiscriminately used and are being permitted in developing countries such as India. These chemicals are highly persistent with a half-life of 3 to 5 years, can bioaccumulate, and have been detected in human breast milk,^3^ drinking water,^4^ ground and river water,^4^ human tissue,^5^ and hair, with higher concentrations among females.^6^ Community practices in India, such as increased burning of waste materials (including plastic and electronics) and crop residue along with use of inadequately recycled or reclaimed water from wastewater treatment plants for agricultural purposes, may lead to higher level of PFAS exposure through contaminated air, soil, and crop foods. Furthermore, the fertilizers, pesticides, and insecticides containing plasticizer formulations used in the agricultural fields when burnt along with the residual crops results in increased level of chemicals in the environment.

PFAS are potentially associated with endocrine and immune dysfunction (including reduced vaccine antibody response) leading to initiation and progression of reproductive and developmental disorders, and early-onset of chronic conditions such as thyroidism, obesity, diabetes, and cancer among children and adults.^7^ These chemicals can cross the placental barrier and perinatally expose the developing fetus to their toxic effects leading to physiological distress to both mother and the offspring.^8^ Although there are no available data on the production, distribution, and use of PFAS in India, a preliminary survey has indicated that though few perfluorooctanoic acid-free cookware are available in the Indian market, they may not be PFAS-free.^9^

In addition to identifying PFAS-containing sources of exposure, which are limitless, as these chemicals are worryingly used in every industry and consumer products, including as plasticizers in production of ultra-processed food, plastic and rubber, high-quality toxicological, and epidemiologic studies are required to be conducted in India for quantifying, evaluating the effects and mechanism of these chemicals implicated in the development of early-onset chronic diseases, especially health concerns associated with women and children. Moreover, programs on ecological and human biomonitoring of PFAS in children and adults need to be initiated for generating valuable evidence for population-wide policy advocacy. Scalable interventions for increasing public and political awareness along with behavioral change at the individual and population-level for restricting availability and use of products containing PFAS could help in reducing persistent exposure.
